# Data-Driven Dynamic Motion Planning for Practical FES-Controlled Reaching Motions in Spinal Cord Injury

**DOI:** 10.1109/TNSRE.2023.3272929

**Published:** 2023-05-11

**Authors:** Derek N. Wolf, Antonie J. van den Bogert, Eric M. Schearer

**Affiliations:** Department of Mechanical Engineering, Vanderbilt University, Nashville, TN 37240 USA; Center for Human-Machine Systems, Cleveland State University, Cleveland, OH 44115 USA; Center for Human-Machine Systems, Cleveland State University, Cleveland, OH 44115 USA, also with the Cleveland Functional Electrical Stimulation Center, Cleveland, OH 44106 USA, and also with the Department of Physical Medicine and Rehabilitation, MetroHealth Medical Center, Cleveland, OH 44109 USA

**Keywords:** Trajectory optimization, neuroprostheses, neuromuscular stimulation, data-driven modeling

## Abstract

Functional electrical stimulation (FES) is a promising technology for restoring reaching motions to individuals with upper-limb paralysis caused by a spinal cord injury (SCI). However, the limited muscle capabilities of an individual with SCI have made achieving FES-driven reaching difficult. We developed a novel trajectory optimization method that used experimentally measured muscle capability data to find feasible reaching trajectories. In a simulation based on a real-life individual with SCI, we compared our method to attempting to follow naive direct-to-target paths. We tested our trajectory planner with three control structures that are commonly used in applied FES: feedback, feedforward-feedback, and model predictive control. Overall, trajectory optimization improved the ability to reach targets and improved the accuracy for the feedforward-feedback and model predictive controllers (*p* < 0.001). The trajectory optimization method should be practically implemented to improve the FES-driven reaching performance.

## I. Introduction

Functional electrical stimulation (FES) neuroprostheses are a promising technology for restoring reaching functions to individuals with upper-limb paralysis caused by spinal cord injury (SCI). Many different techniques including reinforcement learning [1], [2] and iterative learning control [3] have shown success in controlling a single or a couple degrees of freedom in the arm.

These limited successes controlling FES-driven reaching motions have not carried over to individuals with SCI due to the unique actuation issues in their arms. Individuals with SCI suffer from rapid muscle atrophy [4] which combines with increased muscle fatigue when electrically stimulated [5]. Additionally, some muscles suffer a complete loss of function even in the presence of stimulation because of lower motor neuron damage [6] as well as changes in muscle spasticity. While reaching controllers have been implemented in simulation [7], [8], in a rehabilitation setting with people who have suffered a stroke [3], or with healthy individuals [9], the person-specific actuation issues in SCI must be accounted for when controlling practical full-arm reaching motions.

A common method of compensating for the issues in muscle actuation driven by FES is to use a robot to support the desired motions and simplify the complexity of controlling multiple degrees of freedom. For full-arm reaching motions with FES, two seminal approaches are the MUNDUS [10], [11] and BrainGate2 studies [12] which both compensated for these muscular actuation difficulties by using a robot. The MUNDUS project saw major hand position errors arise from slipping in the locked joints, mainly shoulder rotation. For the BrainGate2 system, the major errors in control arose due to the uncontrolled coupled motions produced in other degrees of freedom by the robotic arm support. To better control the arm through reaching motions, it is necessary to develop a controller that directly accounts for the actuation limitations in an individual with SCI as well as the dynamics of the system.

To our knowledge, there have been three main attempts to control full-arm reaching motions without robots actively controlling degrees of freedom [9], [13], [14]. In monkeys, non-disabled muscle activations during reaching were recreated with FES [14], but non-disabled activations may not correspond to a person with SCI. Model-learning methods have been used to determine configuration dependent models of forces produced by the muscles along with a feedback controller to move the hand along a straight-line path to a desired hand position [9], [13]. Razavian achieved 2D reaching motions using FES in a healthy individual [9]. Our own work previously achieved 3D reaching motions using straight-line paths in a participant with a spinal cord injury with reasonable accuracy, but there were areas of the workspace with limited accuracy [15].

Dynamic programming and trajectory optimization have been demonstrated in lower-limb movements as a solution for overcoming these detrimental actuation issues. Simulation studies have demonstrated the need to find feasible trajectories that account for the muscle characteristics caused by SCI [16], [17]. For example, another simulation study found significantly different optimal trajectories for non-disabled walking compared to FES-driven walking trajectories with muscle characteristics of someone with SCI [18]. These studies activated fewer muscles or estimated weakened muscle parameters to simulate the capabilities of an individual with SCI.

The muscle capabilities for every individual with spinal cord injury are unique, and dynamic trajectory optimization must include person-specific capability data to find feasible trajectories. Experimental methods have previously been developed to measure and predict the muscle torque production capabilities of stimulating the arms of individuals with SCI [9], [19]. Combining these models with trajectory optimization to find feasible reaching trajectories has the potential to improve upper-limb control and successfully reach all parts of the workspace. Therefore, we developed a subject-specific, data-driven trajectory optimization that used experimentally measured muscle capabilities to find feasible reaching trajectories.

## II. Methods

**Fig. 1.**
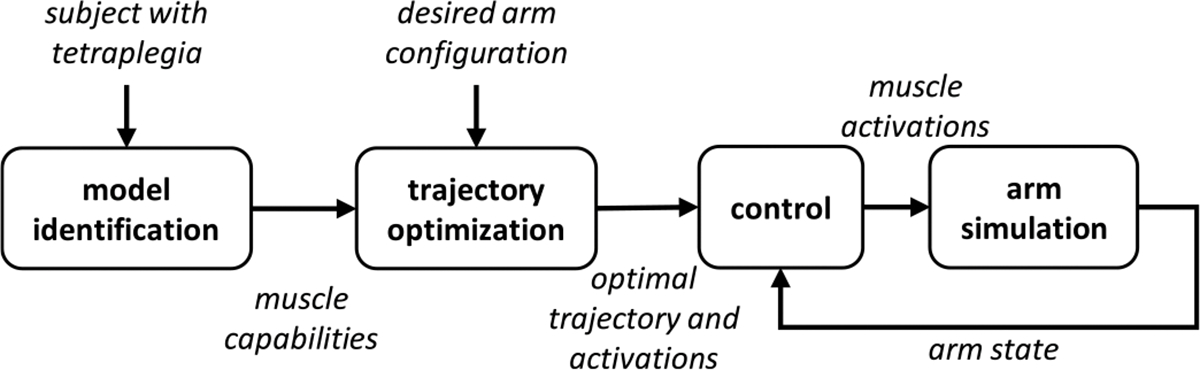
Framework for our control structure presented in this paper. We identify a person-specific model of an arm and its response to electrical stimulation. We then use this model of the muscular capabilities of the participant and a simulation of the arm to find optimal trajectories to achieve a desired arm configuration. Our controller then attempts to drive the arm along the desired trajectory to the target configuration.

We developed a data-driven trajectory optimization method for reaching motions that accounts for person-specific muscle weakness and loss of function. We first identified a person-specific mathematical model of the arm of an individual with high tetraplegia due to SCI and its response to electrical stimulation. We created a dynamic simulation of the arm with the modeled muscle capabilities. We developed a trajectory optimization routine which accounts for the muscle capabilities of the individual and the dynamics of the arm to find feasible trajectories to a target arm configuration. We compared the performance of controlling the arm along these optimized planned trajectories compared to naive direct-to-target paths using three control structures that are commonly used in FES-driven reaching: a feedback controller [9], [15], a feedforward-feedback controller [20], and a model predictive control (MPC) controller [21]. An illustration of our control framework is seen in Fig. 1. The trajectory optimization and simulated control experiments were completed using an HP Spectre laptop with an Intel i7–8565U 1.80 GHz processor.

Informed consent was obtained and the protocols were approved by the institutional review boards at Cleveland State University (IRB NO. 30213-SCH-HS) and MetroHealth Medical Center (IRB NO. 04-00014).

### A. Person-Specific Muscle Model

Our model identification procedures are based on those presented in [22] and [23]. We present a brief summary of the procedure and resulting model here.

**Fig. 2.**
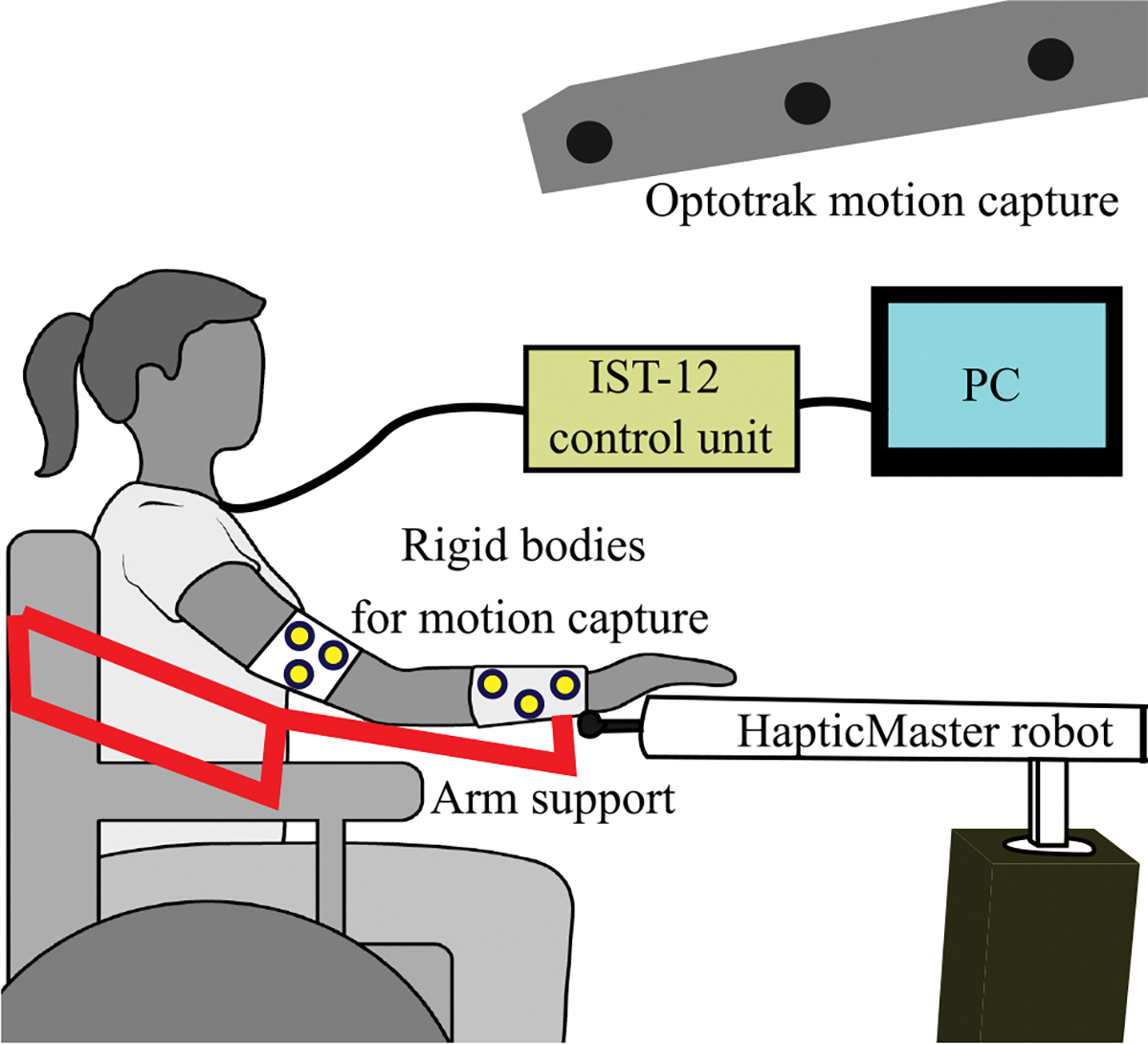
Experimental setup used to identify a person-specific model of an individual’s arm and its response to stimulation.

We completed a system identification experiment with a single human participant with high tetraplegia. The individual sustained a hemisection of the spinal cord at the C1-C2 level. We worked with her right arm which she is unable to voluntarily move except for limited shrugging of the shoulder. She exhibits normal to hypersensitive sensation on her right side and does exhibit hypertonia in some of her muscles. More details on the participant can be found in [24] (participant 1). The experimental setup is shown in Fig. 2.

The individual is implanted with the IST-12 stimulator telemeter in her abdomen [25]. We controlled nine muscle groups with the device: 1. triceps (electrode type: nerve cuff, maximum pulse-width: 250 *μ*s, current amplitude: 2.1 mA), 2. deltoids (nerve cuff, 23 *μ*s, 2.1 mA), 3. latissimus dorsi (nerve cuff, 10 *μ*s, 0.8 mA), 4. serratus anterior (nerve cuff, 20 *μ*s, 1.4 mA), 5. biceps and brachialis (nerve cuff, 49 *μ*s, 0.8 mA), 6. supraspinatus and infraspinatus (62 *μ*s, 1.4 mA), 7. rhomboids (intramuscular, 107 *μ*s, 18.0 mA), 8. lower pectoralis (intramuscular, 22 *μ*s, 18.0 mA), and 9. upper pectoralis (intramuscular, 25 *μ*s, 20.0 mA). Muscle stimulation uses bi-phasic, charge balanced pulses delivered at 13 Hz. The maximum stimulation parameters are defined for subject comfort and safety.

To gather data for model-learning, we used a HapticMaster (Moog FCS) robot with three degrees of freedom. The robot records the 3D forces at its end-effector. The participant’s wrist was attached to the robot via the ADL gimbal attachment (Moog FCS) which transmits force but not torque to the robot. An Optotrak Certus Motion Capture System (Northern Digital, Inc.) captured data used to calculate the arm’s configuration defined as three rotations at the shoulder — shoulder plane of elevation, shoulder elevation, and shoulder rotation — and two rotations at the elbow - flexion and pronation — as defined by the ISB standards [26]. (For reference, the standard anatomical motion of shoulder abduction/adduction corresponds to shoulder elevation at a 0-degree plane of elevation. The standard anatomical motion of shoulder flexion/extension corresponds to shoulder elevation at a 90-degree plane of elevation.) At 27 positions spaced throughout the participant’s comfortably reachable workspace — defined as the space in which the robot can comfortably move the subject’s hand — we measured the amount of force produced by each muscle group when stimulated at their maximum pulse-width as well as with no muscle groups stimulated with the wrist held statically by the robot. When multiplied by the transpose of the Jacobian of the arm, the torques about each of four degrees of freedom — shoulder elevation plane, shoulder elevation, shoulder rotation, and elbow flexion (pronation does not create force at the wrist) — can be calculated. (The force capabilities of the subject’s muscles are visualized in [19], and representative force and torque measurements during model identification are shown in the supplemental Fig. S1 and S2.) The process was repeated three times, and the data was used to train a semiparametric Gaussian process regression (GPR) model [27] for each muscle group. The input to the model is the configuration of the arm and the output is the joint torque predicted to be measured by the robot when a muscle group is stimulated. The difference between the predicted torque with no muscles stimulated and with a muscle group stimulated is the predicted amount of torque produced by the muscle group.

It is assumed that the torques produced by the muscle groups combine linearly, an assumption that is supported by [28]. Therefore, the torque, $\tau \in R^{4}$ produced by a set of muscle activations, $\alpha \in R^{9}$ where α∈[0,1] for each muscle group, is determined by

(1) τ=R(q)α

where q is the arm configuration and $R(q)\in R^{4\times 9}$ is the configuration dependent muscle torque production matrix. The $i^{\text{th}}$ column of R corresponds to the torques produced by the $i^{\text{th}}$ muscle group when stimulated at 100% activation.

Using the semiparametric GPR model, the capabilities of the individual’s muscles at any arm configuration in their workspace can be predicted by calculating the muscle torque production matrix.

### B. Dynamic Arm Simulation

We developed a dynamic simulation of the participant’s arm that used our previously found muscle torque production models to simulate the participant’s true capabilities. The simulation consisted of two links, a humerus and a forearm, and four degrees of freedom. There were three rotations at the shoulder: shoulder plane of elevation, shoulder elevation, and shoulder rotation. There was one degree of freedom for elbow flexion. All rotations are defined in [26].

The segment lengths of the participant were measured to be 0.315 m for the humerus and 0.253 m for the forearm. The mass, moments of inertia, and position of the center of masses for each link were estimated using the properties from [29]. The equations of motion were found using Autolev 4.3 [30]:

(2) $$\tau =M(q)\ddot{q}+C(q,\dot{q})\dot{q}+g(q),$$

where M(q) is the mass matrix, $C(q,\dot{q})$ is the Coriolis matrix, and g(q) is the gravitational and passive stiffness term. For numerical stability, the simulation included stiffness of 1 Nm/rad and damping of 1 Nms/rad on each degree of freedom with the equilibrium configuration being the passive equilibrium measured with the participant. The equations of motion were rearranged into implicit state space form,

(3) $$f(x,\dot{x},\alpha )=0,$$

where the state, x, incldues the angles and angular velocities: $x=(q,\omega {)}^{⊤}$, and

(4) $$f=\left[\begin{array}{c} \dot{q}-\dot{\omega }\\ M\left(q\right)\dot{\omega }+C\left(q,\dot{q}\right)\omega +g\left(q\right)-R\left(q\right)\alpha \end{array}\right].$$

Symbolic differentiation was used to generate C code for the function f, and its three Jacobian matrices $-df/dx,df/d\dot{x},\;df/d\alpha$ — to allow gradient-based trajectory optimization and linearization. The C code was compiled into a MEX function for use in MATLAB.

The model was actuated using torques across each of the degrees of freedom. The inputs to the model were the set of nine muscle activations, and equation (1) was used to determine the torque across each joint.

The simulation made several assumptions regarding the dynamics of the real-life system. The simulation did not include gravity. The participant’s shoulder muscles are not strong enough to support against the force of gravity. Due to this, the participant uses a mobile arm support to support against the force of gravity. We assumed that the arm support perfectly compensates for the force of gravity on the arm.

The system was simulated using the backwards Euler method with a time step of 0.02 seconds. Newton’s method was used to find the next state of the system at the end of the time step [29], [31]. For each time step, the control inputs were discretized and held constant across the entire time step which is realistic to how the real stimulation systems work where the frequency of stimulation determines the rate at which control inputs can change. The dynamics of the system and the muscle torque production models were modeled as continuous systems which varied with the state of the system, defined by the joint angles and joint velocities of the arm. The system was simulated using MATLAB r2019b (The MathWorks, Inc).

### C. Trajectories

#### 1) Direct-to-Target Trajectories:

Direct-to-target trajectories were defined as a similar approach to the straight-line trajectories followed in previous research [9], [15]. We defined a fifth-order polynomial for each joint which began at the starting arm configuration with zero velocity and ended at the target configuration with zero-velocity producing smooth, minimum-jerk trajectories similar to natural human reaching [32].

#### 2) Optimized Planned Trajectories:

Using the dynamic arm simulation presented in II-B as a basis, trajectory optimization was used to determine feasible reaching trajectories that accounted for the person-specific muscle capabilities. We attempted to find a feasible trajectory from a given starting configuration to a target configuration. From (1), we can see that for some target configurations, it was possible that no feasible trajectory exists because the modeled muscles are unable to produce torque in the required direction. To search for a feasible trajectory, we used the trajectory optimization techniques described for optimizing human gait in [33]. We used IPOPT [34] to solve a direct collocation constrained nonlinear optimization problem to calculate the optimal muscle activations to achieve the desired motion.

With known dynamics from (4), for n total nodes, the trajectory optimization problem was written as

(5) $$\underset{\alpha ,x}{minimize}\;mean\left(\alpha^{2}\right)+\gamma \;mean({\left(q-q_{\text{targ}\;}\right)}^{2})\;\text{subject}\;\text{to:}\;\;\text{state}\;\text{constraints}\;\;x_{min}\leq x_{k}\leq x_{max},\;\forall k\in \{1,\;2,\ldots ,n\}\;\;\text{activation}\;\text{constraints}\;\;\alpha_{i,k}\;\in [0,\;1],\forall i\;\in \{1,\;2,\ldots ,9\},\;\forall k\in \{1,\;2,\ldots ,n\}\;\;\text{dynamics}\;\text{constraints}\;\;f\left(x_{k},{\dot{x}}_{k+1},\alpha_{k}\right)=0,\;\forall k\in \{1,\;2,\ldots ,n-1\}\;\;\text{task}\;\text{constraints}\;\;x_{1}={\left[q_{0}0\right]}^{⊤}\;x_{n}={\left[q_{targ}0\right]}^{⊤}.$$

The first term of the objective function minimizes the average of the squared muscle activations for all n nodes of the trajectory, α. Minimizing muscle activations is desirable because it limits fatigue in the participant and allows for greater control bandwidth for a feedback controller to adjust activation before muscle saturation. The second term attempts to minimize the average distance from each configuration across all n nodes of the trajectory, q, to the final target configuration, $q_{\text{targ.}}$. This term was added to bias the solution to more direct paths. While paths that go directly to the target are not always reasonable, for most reaching tasks, a person will want to reach in the most direct path possible to achieve the desired motion. γ is a weighting factor which was selected to be $\gamma =1{rad}^{-2}$ to achieve the overall goal of the objective function to balance the goals of minimal activations and direct path reaches.

The optimization problem includes constraints on the state (joint angles and joint velocities), muscle activations, dynamics, and task. To guarantee the controller found trajectories within the participant’s comfortable workspace, the joint angles were constrained to be between the minimum and maximum joint angles seen during the system model identification in section II-A with an additional 11° of rotation in each direction to ensure trajectories along the edge of the workspace could be reached. The joint velocities had a maximum magnitude of 10 rad/s. The combined state constraints are represented by $x_{min}$ and $x_{max}$. The muscle activations were constrained between 0 and 1. The dynamics constraints ensured that the dynamics from the simulation developed in section II-B were satisfied throughout the trajectory. The dynamics were approximated using the backward Euler method. The task constraints ensured that the first node began at the start configuration with zero velocity, $x_{0}$, and the final node ended at the target configuration with zero velocity, $x_{\text{targ}}$.

To select the target arm configurations, we created a grid of arm configurations with 20° spacing between the maximum and minimum joint angles measured in the training data in section II-A. This resulted in a grid of 81 target configurations — and corresponding 3-dimensional hand positions — spaced throughout the subject’s workspace (see Fig. 5a). The desired starting configuration for each reaching motion was defined as the resting equilibrium configuration as measured while identifying the model. This configuration placed the participant’s wrist near the center of their reachable workspace. For each target configuration, we completed the trajectory optimization with 100 nodes (time-step of 0.02 s). Increasing the number of nodes increases the computational load but improves the estimation of the system dynamics. To select the number of nodes, an optimization was completed for a single trajectory using 200 nodes, and this optimized trajectory was accepted as the ground truth. We then completed a series of optimizations using different numbers of nodes, and the root mean squared (rms) error of the predicted trajectory to the ground truth trajectory was calculated. We started with ten nodes and increased the number of nodes until the new trajectory had a final rms error of 1 mm when compared to the 200-node trajectory.

The duration of each trajectory was 2 seconds. For the first attempt at finding a trajectory for a given target position, we used an initial guess of a direct-to-target trajectory (fitting a fifth-order polynomial for each joint from the start to the target arm configuration) with zero activation. If IPOPT was unable to find an acceptable solution in 1500 iterations, we would try to find a feasible trajectory for the target position one additional time with a random initial guess. If a feasible trajectory was still not found, the target position was abandoned and the next target configuration was attempted. It is important to note that not finding a feasible trajectory does not guarantee that one does not exist. For all targets, the amount of time to complete the optimization ranged from 11 seconds to 826 seconds with an average time of 323 seconds. For feasible targets, the average amount of time to complete the optimization routine was 79 seconds.

### D. Controlling Simulated Reaching Motions

To test our trajectories found with the optimization methods in II-C.2, we compared three controllers: 1. a feedback controller (referred to as “ FB” in figures and tables), 2. a combined feedforward-feedback controller (“FF+FB”), and 3. a model predictive control (“MPC”) controller.

#### 1) Feedback Controller:

The feedback controller is similar to the controller presented in [15] used for straight-line reaching. A PID controller transforms errors in joint-position and velocity to desired control torques, $\tau_{FB}\in R^{4}$, across each degree of freedom. For the current configuration of the arm, q, the muscle torque production matrix, R(q), is predicted using the person-specific model developed in II-A. For the feedback only controller, to resolve muscle redundancy and solve for the desired muscle activations, α, we then solve the following quadratic programming problem,

(6) $$\begin{array}{l} \underset{\alpha }{minimize:}∥\alpha {∥}_{2}^{2}\\ \;\text{subject}\;\text{to:}\;R(q)\alpha =\tau_{FB}\\ \;\alpha_{i}\in [0,\;1]\;\forall i\in \{1,\;2,\ldots ,9\}. \end{array}$$

If overcompensation oocurs, meaning the feedback controller calls for torques which are infeasible due to the muscle capabilities of our participant, the controller attempts to find the muscle activations which produce the maximum torque in the desired direction of $\tau_{FB}$. We achieve this by asking for 70% of the requested torque. If no feasible solution is found with the new requested torque, we continue to scale down the requested torque to 70% of the previous requested torque to find a set of muscle activations which produce torque in the desired direction. If after 10 iterations no solution is found, the controller outputs zero muscle activation.

The parameters of the PID controller were manually tuned on several trajectories with the goal of producing accurate reaches with smooth activation profiles. For the feedback controller, the proportional gain was 10 N/rad, derivative gain was 1 N-s/rad, and integral gain was 1 N/rad-s.

#### 2) Feedforward-Feedback Controller:

For the feedforward-feedback controller, the feedforward activations, $\alpha_{ff}$, were derived from the trajectory optimization for the planned trajectories. For the direct-to-target trajectories, we solved the inverse dynamics problem using a nonlinear interior-point optimization to find the feedforward muscle activations that would drive the dynamic arm simulation along the desired trajectory. The feedforward-feedback controller used the same PID controller to transform errors in joint-position and velocity to desired feedback control torques, $\tau_{FB}\in R^{4}$, across each degree of freedom as described above. The feedforward activations were added to the activation commands produced by the feedback control, $\alpha_{fb}$, and ensure that the total activation for each muscle is between 0 and 1. The new optimization problem becomes

(7) $$\begin{array}{l} \underset{\alpha_{fb}}{\mathrm{minimize:}}{\left\|\alpha_{\text{fb}}\right\|}_{2}^{2}\\ \text{subject}\;\text{to:}\;R(q)\alpha_{\text{fb}}=\tau_{\text{FB}}\\ \;\alpha_{\text{fb},i}+\alpha_{\text{ff},i}\in [0,1]\;\;\forall i\in \{1,2,\ldots ,9\}. \end{array}$$

The same overcompensation strategy as presented above is used to select the feedback muscle activations. If feasible activations cannot be found, the feedback activations are set to zero and only the feedforward activations are used. The overall muscle activations applied to the arm are $\alpha =\alpha_{\text{fb}}+\alpha_{\text{ff}}$.

The PID controller parameters of the feedforward-feedback controller were kept the same as the feedback controller for consistency: the proportional gain was 10 N/rad, derivative gain was 1 N-s/rad, and integral gain was 1 N/rad-s.

#### 3) MPC Control:

We also developed an MPC control scheme with the hypothesis that including knowledge of the system dynamics more explicitly in the controller would produce more accurate reaches. Additionally, MPC controllers are able to explicitly account for the constraints of the system and thus eliminate the issue of overcompensation. The MPC control scheme we developed is based on the incremental MPC formulation presented in [35] which incorporates the benefits of integral control to the MPC control scheme. To find the continuous time state-space matrices of the system, $A_{c}$ and $B_{c}$, the Jacobians of the equations of motion found in II-B were used to linearize the system about the current state,

(8) $$\begin{array}{r} A_{c}=-\frac{df^{-1}}{d\dot{x}}\frac{df}{dx}\\ B_{c}=-\frac{df^{-1}}{d\dot{x}}\frac{df}{d\alpha }. \end{array}$$

The state-space system was discretized using a zero-order hold. The state of the system included the joint angles and joint velocities. The output of the system and the reference trajectory included only the joint angles.

At each time-step, k, the discretized state-space model of the system can be written as

(9) $$\begin{array}{l} x_{k+1}\;=Ax_{k}+B\alpha_{k}\\ \;y_{k}\;=Cx_{k}, \end{array}$$

which can be used to predict the next state of the system, $x_{k+1}$, and the current system output, $y_{k}$. To add integral action to the controller, the state is augmented with the current control input, and the new control input is defined as the change in control input, Δu. The state-space system becomes

(10) $$\begin{array}{c} \left[\begin{array}{c} x_{k+1}\\ \alpha_{k} \end{array}\right]\;=\left[\begin{array}{cc} A & B\\ 0 & I \end{array}\right]\left[\begin{array}{c} x_{k}\\ \alpha_{k-1} \end{array}\right]+\left[\begin{array}{c} B\\ I \end{array}\right]\Delta \alpha_{k}\\ y_{k}\;=\left[\begin{array}{ll} C & D \end{array}\right]\left[\begin{array}{c} x_{k}\\ \alpha_{k-1} \end{array}\right]+D\Delta \alpha_{k-}. \end{array}$$

These state-space matrices are assumed constant for the control calculations during a given time-step. The controller selects the commands which minimize the objective function

(11) $$J=\sum_{i=1}^{n_{y}}e_{k+i}{}^{T}e_{k+i}^{T}e_{k+i}+\lambda \sum_{i=0}^{n_{u}-1}\Delta \alpha_{k+i}{}^{T}\Delta \alpha_{k+i}.$$

The first term of the equation minimizes the error, $e_{k+i}$, for a given time-step which is defined as the estimated output as calculated by (10) subtracted from the reference trajectory. The prediction horizon, $n_{y}$, determines for how many time steps forward the model predicts states and system error. The control horizon, $n_{u}$, determines the number of time steps forward that the controller optimizes control inputs. For time steps $n_{\alpha }<i<n_{y},$ Δu=0. The lumped scalar weighting λ acts as a muscle group activation smoothness parameter by weighting the amount that the activation commands change.

The parameters of the MPC controller were tuned on several trajectories with the goal of producing accurate reaches along with smooth activation profiles. The time step of the simulation was 0.02 seconds. The prediction horizon was selected to be four time steps, and the control horizon was two time steps. This weighting on the change in muscle activations, λ, was selected to be 0.001 which was the highest value that did not see a large drop in accuracy. We selected this highest value of the control input weighting to create smoother activation profiles which are more comfortable.

### E. Simulation Experiments

We tested a set of 30 reaches from a neutral starting position to target arm configurations as found by the trajectory optimization in section II-C.2. For each target reach, we tested all combinations of planned and direct-to-target paths, controllers, and variations of model uncertainty described below.

#### 1) Planned vs Direct-to-Target Trajectories:

To evaluate the importance of trajectory planning to control FES-driven arms of individuals with SCI, we controlled the simulated arm following the planned trajectories found in section II-C.2 and naive direct-to-target trajectories as defined in II-C.1.

#### 2) Control Strategy:

We compared three control strategies for driving the arm along the desired trajectory. We used the feedback, feedforward-feedback, and MPC controllers presented in section II-D to drive the arm along each trajectory. For the direct-to-target trajectories driven by the feedforward-feedback controller, the feedforward commands were found by solving the inverse dynamics with the known arm simulation model. Feedforward commands found during trajectory optimization were used during the feedforward-feedback control of planned trajectories. For feedback and MPC control of trajectories, the controllers attempted to drive the arm along the planned trajectory without any feedforward control inputs.

#### 3) Model Uncertainty:

We first used an ideal model where the controller had perfect information about the dynamics of the system (i.e., the muscle model in the controller perfectly matched the muscle model of the dynamic simulation).

To demonstrate our methodology for later real-world application with human participants, each controller was tested with an “uncertain model”. The muscle torque production capability model used in the arm simulation that we were controlling was different than the muscle torque capability model used by the controller. The uncertain muscle models were created by developing a new set of training data for the models produced tin section II-A. The training data were randomly pulled from the predicted distribution (mean and variance) calculated by the semiparametric GPR models. We repeated the control with uncertain models for all trajectories 10 times to ensure a wide selection of uncertain muscle capability matrices. In a real-world scenario, model errors lead to muscle capability predictions of both incorrect magnitude and incorrect amount of torque on each degree of freedom (joint-space torque direction vector). This uncertain model would similarly produce changes in both the magnitude and direction of torque produced by each muscle group.

Another realistic scenario is the presence of fatigue which affects the magnitude of torque created but not the direction. To test the controller’s response to fatigue, a “fatigued model” was created by limiting the magnitude of the muscle torque production matrix of the arm simulation to 90% of the predicted value used by the controller.

#### 4) Data Analysis:

To measure the accuracy of the controllers, we defined the error of a given reach in joint-space as the Euclidean distance from the final arm configuration and the desired configuration and in Cartesian space as the 3-dimensional Euclidean distance from the final hand position to the target hand position. While the controllers drove the arm in joint-space, for an individual person, the most important measure for completing functional tasks is the ability to place the participant’s hand at a target; therefore, we use hand position as the main measurement of success. The main outcome metrics of the control experiments were: 1) the number of targets where the final error was at most 5 cm and 2) the average error across all 30 targets.

The main goal of the paper was to determine the necessity of motion planning. We compared the two outcome metrics (targets reached with 5 cm error and overall average error) within the trials for each controller driving the arm along a planned trajectory vs. a direct-to-target path. Kruskal-Wallis tests were completed to compare the average error results of these same controller comparisons.

The analysis was then repeated using the results from the controllers using the uncertain and fatigued models.

## III. Results

Using a person-specific model of an individual with SCI’s arm driven by FES, we used trajectory optimization to find feasible reaching trajectories from a starting position in the center of the workspace to target arm configurations throughout the participant’s reachable workspace. Out of a grid of 81 potential target arm configurations, we found 30 feasible trajectories from a central equilibrium position to target positions throughout the participant’s workspace (see Fig. 5a for an image of the feasible target positions). Reaches ranged from 2 to 36 cm in length with an average reach length of 20 cm. For each trajectory, we simulated three controllers: feedback, feedforward-feedback, and MPC to drive the arm along both naive direct-to-target paths and planned trajectories found using trajectory optimization.

**Fig. 3.**
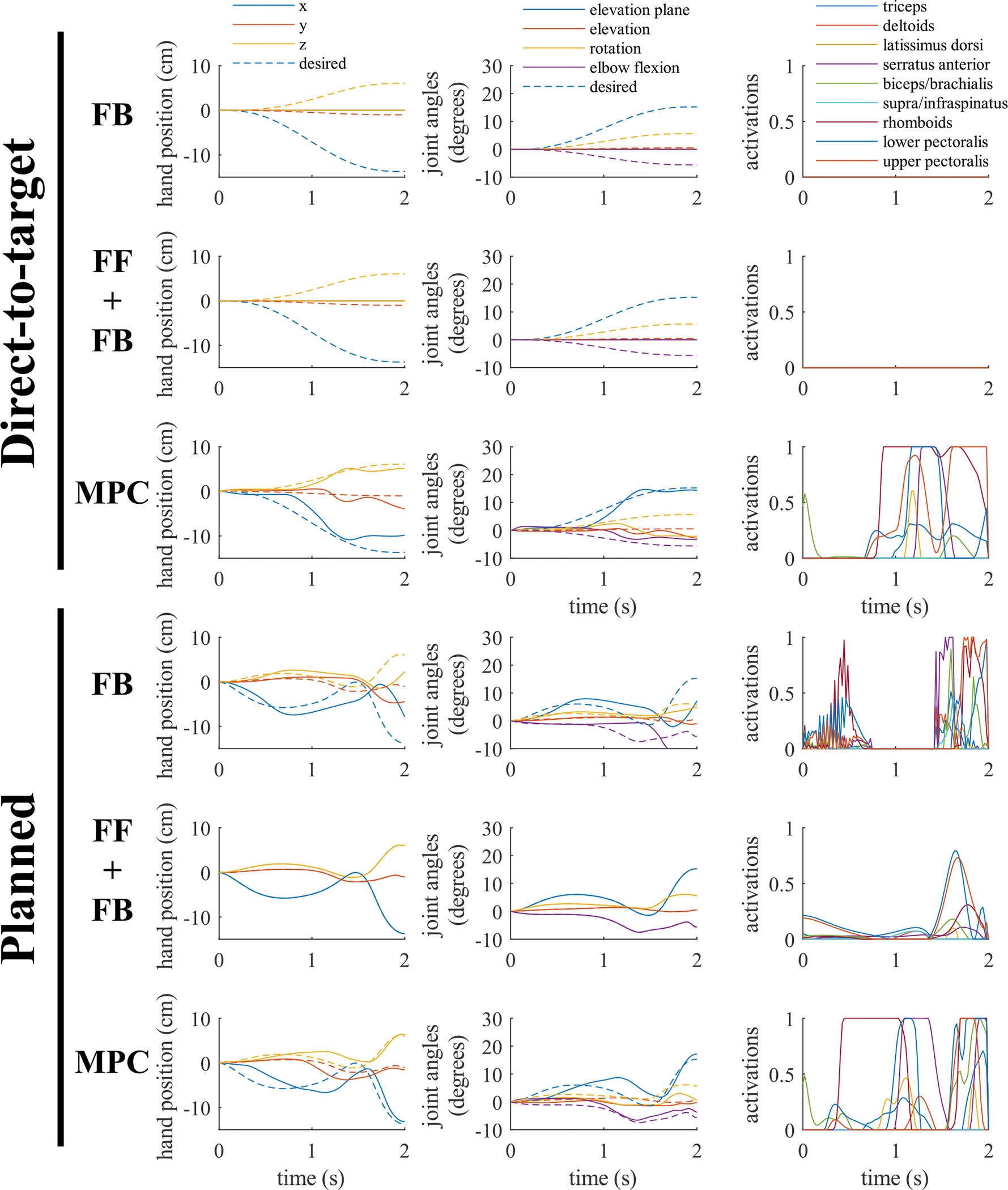
A representative reach showing the hand position, joint angles, and activation patterns for controlling a simulated arm when controlled along direct-to-target and planned trajectories for the same target with an ideal model for the feedback (FB), feedforward-feedback (FF+FB), and MPC controllers. To better show the movement of the hand and all four joints, the hand position and joint angles are plotted relative to the starting position and configuration respectively. The feedforward-feedback and feedback controllers are unable to produce torque to travel along the direct-to-target path. However, the MPC controller is able to deviate from the path in some joints to perform a more accurate reach by driving better following the shoulder elevation plane.

A representative example of a set of reaches for a single target configuration is shown in Fig. 3. This is a 15 cm reach and the hand position trajectory is shown in Fig. 5a. When driving the arm along direct-to-target trajectories, the feedback controller produced an error of 15.0 cm, the feedforward-feedback controller had an error of 15.0 cm, and the MPC controller achieved an error of 5.0 cm. For directto-target trajectories, the feedforward-feedback controller and feedback controllers performed identically because the inverse dynamics was unable to find activations to achieve the desired direct-to-target trajectory resulting in zero feedforward activations. This was common for all direct-to-target trajectories and demonstrates the need to plan smarter trajectories that account for the participant’s muscle capabilities. Planning improved all controllers for this representative reach with the feedback controller producing an error of 7.8 cm, the feedforward-feedback controller achieving 0.0 cm of error, and MPC controller achieving an error of 0.9 cm.

The feedback and feedforward-feedback controllers for the direct-to-target trajectory and the feedback controller for the planned trajectory became stuck in configurations in which the controller called for torques in a direction that the muscles cannot achieve (Fig. 3). Therefore, the controller, unable to find activations to produce any torque in the desired direction, requested zero muscle activation, and the arm did not move in the desired direction. The MPC controller, on the other hand, avoided this situation by using knowledge of the dynamics and muscle capabilities of the system to deviate from the desired trajectory for some joints to allow other joints to move closer to the desired target and produce a more accurate reach. Though not seen in this example with an ideal model and planned trajectory, this issue of overcompensation and a feedback controller applying zero torque can also occur in the feedforward-feedback controller in planned trajectories.

**Fig. 4.**
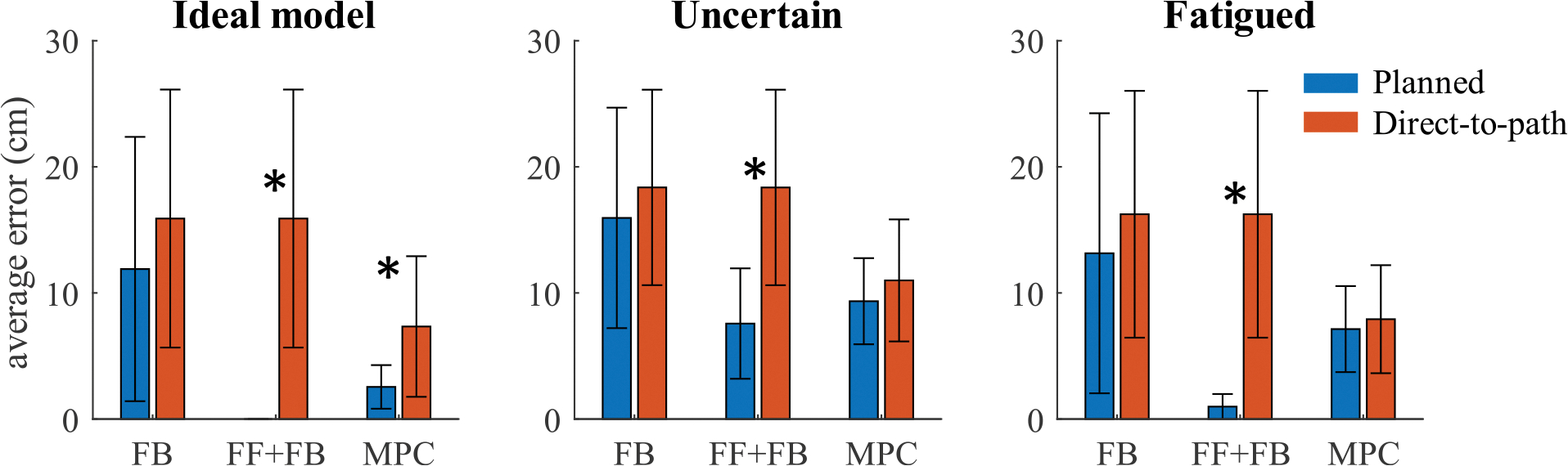
Planning trajectories generally improved the controller accuracy. This figure shows the average error and standard deviation for the planned and direct-to-path trajectories across each controller and for all model conditions. For each controller, a star denotes when trajectory planning results in significantly different error (*p* < 0.05) compared to direct-to-path trajectories.

**Fig. 5.**
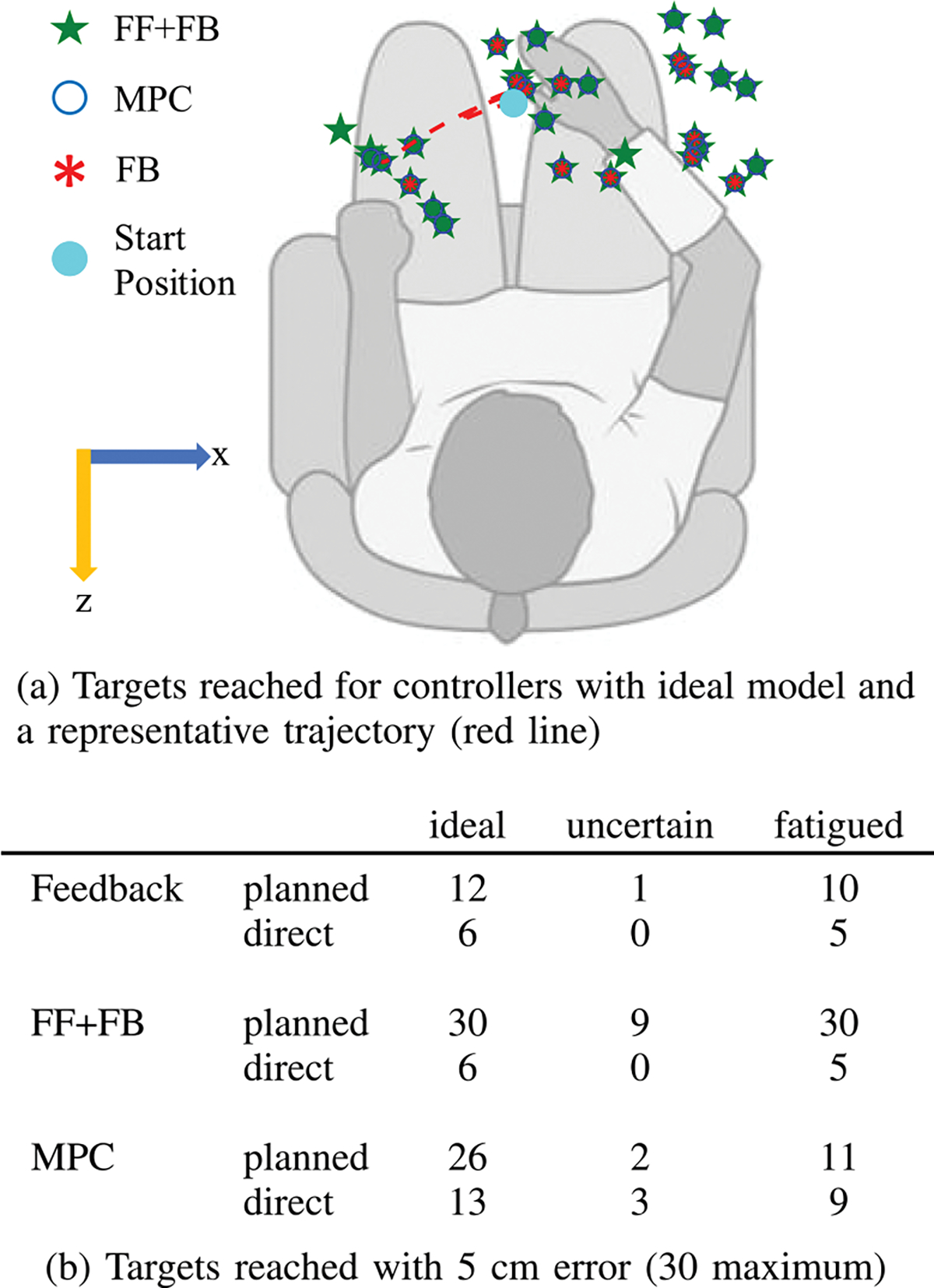
(a) The target hand positions of targets which are achieved with less than 5 cm error using the planned trajectories and each of the three control strategies. The feedforward-feedback (FF+FB) controller achieves all 30 targets, the MPC controller achieves 26 targets, and the feedback (FB) controller achieves 12 target hand positions. (b) This table describes the performance of all control strategies in the number of targets out of a possible 30 that the controller was able to achieve final hand positions with less than 5 cm error when using an ideal model.

**Fig. 6.**
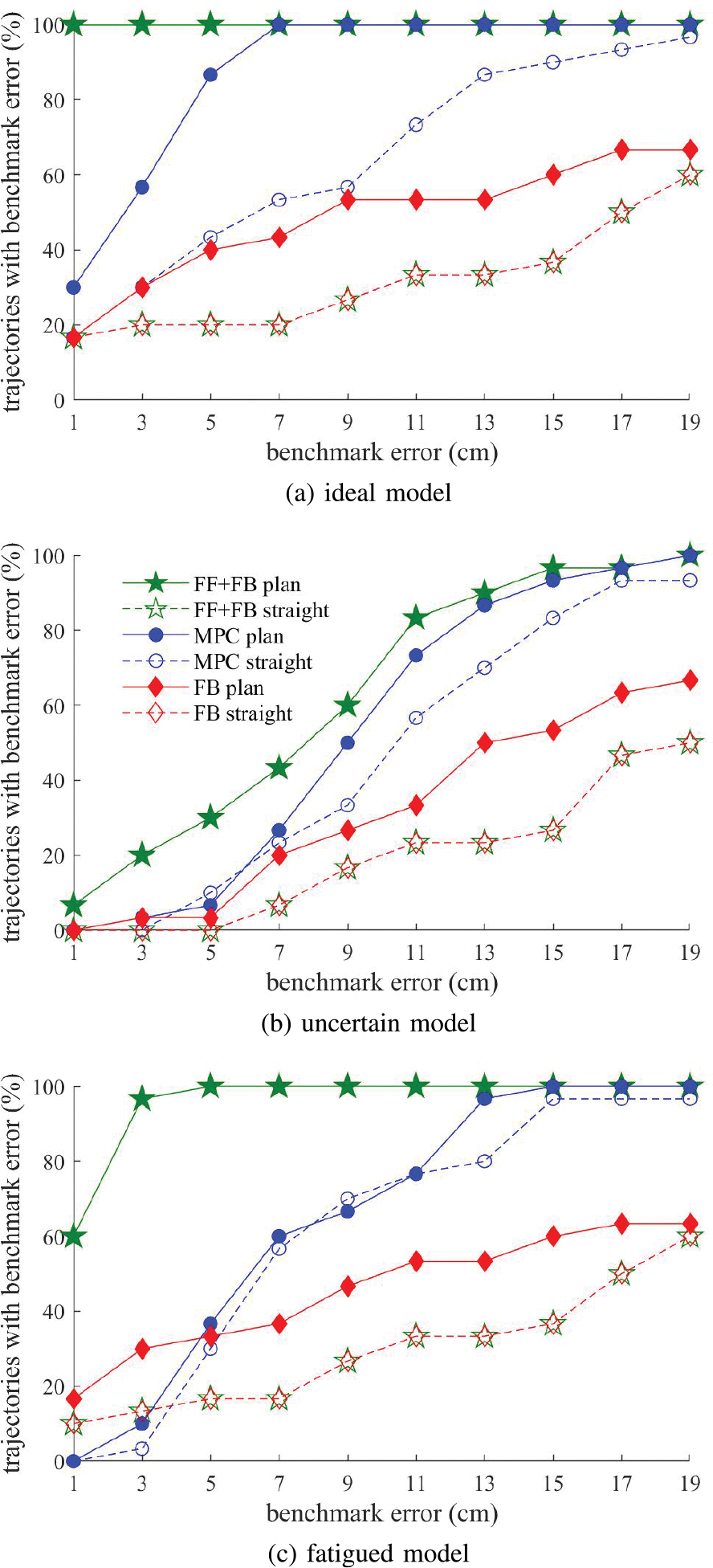
This plot shows the percentage of trajectories (out of 30 attempted reaches) for which each controller achieved less than the benchmark error on the horizontal axis when using a (a) ideal model, (b) uncertain model (average of 10 trials is shown), and (c) fatigued model.

These patterns in performance are seen for all reaches in Fig. 4 and Fig. 5b which show the average final error and the number of targets out of the 30 possible trajectories that were reached with less than 5 cm error for each control experiment respectively. Fig. 6 shows the percentage of targets (out of the 30 possible trajectories) that were reached by each controller and each model uncertainty conditions with less than the benchmark level of error for benchmark errors ranging from 2 to 20 cm.

Overall, for the ideal model, planning generally resulted in improved controller performance. As seen in Fig. 5b and 6a, trajectory planning resulted in an improved ability to reach more positions throughout the individual’s workspace. Following planned trajectories, the feedback controller reached 12 target hand positions with an error of less than 5 cm compared to only 6 target hand positions with direct-to-target paths. The feedforward-feedback controller achieved 30 targets with planned trajectories compared to only 6 for direct-to-target trajectories. For the MPC controller, planning resulted in reaching 26 targets compared to 13 targets without planning. The ability to reach more positions throughout the workspace is critical for achieving daily reaching tasks. Trajectory optimization improved the accuracy for all 30 targets for feedforward-feedback, 25 targets for MPC, and 16 targets for feedback control. The difference in error was significant for the feedforward-feedback controller (*p* < 0.001) and MPC (*p* < 0.001), but not for the feedback controller (*p* = 0.2).

For the uncertain model, planning once again leads to improved performance, however the difference is less pronounced. Planning leads to reaching more targets with 5 cm error for both the feedback and feedforward-feedback controllers (see Fig. 5. At benchmark errors of better than 7 cm, trajectory optimization improves all controllers (6b). Trajectory optimization improved the accuracy for 28 of 30 targets for feedforward-feedback control, 21 targets for MPC, and 11 targets for feedback control. The difference in error was significant for the feedforward-feedback controller (*p* < 0.001) but not for the MPC (*p* = 0.2) or feedback only controller (*p* = 0.9).

For the fatigued model, trajectory planning improves the controller performance for reaching targets with at most 5 cm error (see Fig. 5b). Trajectory optimization improved the accuracy for 29 of 30 targets for feedforward-feedback control, 14 targets for MPC, and 15 targets for feedback control. The difference in error was significant for the feedforward-feedback controller (*p* < 0.001) but not for the MPC (*p* = 0.7) or feedback only controller (*p* = 0.3).

In all model conditions, the feedforward-feedback and MPC controllers generally performed better than the feedback only controller. This is likely due to improved incorporation of the system dynamics to avoid the overcompensation issues as seen in Fig. 3.

## IV. Discussion

We developed a data-driven, person-specific trajectory optimization scheme that accounts for the experimentally measured muscle characteristics of a person with SCI to find feasible reaching trajectories. Combined with commonly used FES-control schemes, this planner resulted in reaching more targets throughout the subject’s workspace. More advanced controllers that incorporate knowledge of the arm’s dynamics to the controller via feedforward activations or in an MPC control strategy may further improve the performance.

Reaching all portions of the participant’s workspace was found to be difficult with straight-line paths in [15], and we were able to recreate that result with the direct-to-target trajectories in this study including demonstrating situations where simple PID feedback controllers will fail to produce activations. As had been observed in [19] and [36], due to the unique muscle capabilities of individuals with SCI the workspace of the person will include configurations that are not controllable in that the muscles are unable to drive the arm in the direction of the next desired state. The need to account for these unique, person-specific capabilities prevents straight-line feedback controllers such as the one presented in [9] from being successfully implemented in individuals with SCI for full-arm 3D reaching motions. Even in the presence of no uncertainty in the controller, if uncontrollable configurations are not avoided and planned for, as seen in this paper, the reaching motion will not be successful. This point is made more clear by the fact that feedforward commands could not be found for direct-to-path reaches using a known model of the system’s capabilities and dynamics. This is because the muscles are unable to produce torque in any desired direction at any given configuration. This point bears repeating, even with an ideal model of the participant’s muscle capabilities and the dynamics of the system, trajectory optimization is necessary to avoid paths which include uncontrollable configurations and produce accurate reaches.

While we attempted to model some level of uncertainty, the dynamic simulation presented in this study is very basic and does not include many nonlinearities and sources of uncertainty which exist in individuals with SCI including electro-mechanical delays, muscle activation dynamics, rapid fatigue, and the nonlinear elasticity of the arm-support. When practically implementing the control framework in an individual with SCI, the increased uncertainty makes it even more critical to avoid uncontrollable configurations, and planning alone may not be able to do so. While the feedfoward-feedback controller produced the best accuracy in this study, there were still situations where the arm would get stuck in an uncontrollable configuration due to uncertainty in the model, and zero feedback muscle activation would be requested for all muscle groups. The use of an MPC controller may offer a solution to this specific issue as the controller is able to determine which degrees of freedom to prioritize to best move towards the desired target. MPC control has been demonstrated to be successful in other FES implementations [37], [38]. However, in this study it did not perform as well overall in the presence of model errors. Tube-base MPC has been developed in lower-limb hybrid exoskeleton control to account for model errors [39]. It is also important to improve the model used in the MPC control. Many of the errors in the controller can most likely be attributed to linearizing the system. To avoid this problem, one method would be to develop a GPR model of the system dynamics directly as presented in [40] and [41].

Other methods of trajectory optimization could also be used to better avoid uncontrollable locations — again, defined as a location where the muscles cannot produce torque in any arbitrary direction — to improve the performance of all controllers. Some of the found trajectories were on the edge of controllability, and even small deviations would lead to large errors. One possible solution to this would be to map the controllability of the configuration workspace. Previous research has attempted to map the configuration dependent capabilities of the workspace for rehabilitation purposes [19]. With a similar mapping, additional terms could be added to the trajectory optimization or a trajectory optimization algorithm such as CHOMP [42] could be used to bias the trajectories away from uncontrollable locations.

The feedback-feedforward controller performed similarly to some other simulation studies with an error of 11.7 degrees (Euclidean distance of all joint angles) in the uncertain condition and 1.2 degrees in the fatigued. Blana, et. al. achieved an rms error of 4 degrees at the shoulder and elbow for 2D reaching with a fatiguing muscle model [20]. Cooman developed a feedforward-feedback controller with time-delay compensation that achieved rms errors ranging from 3.9–10 degrees for each degree of freedom at a maximum of 10% uncertainty in the model’s inertial parameters [43]. However, our controllers accounted for the true muscle capabilities of an individual with SCI, including a limited subset of available muscles, while Cooman’s controller used 24 independent muscles with no uncertainty in the muscle capabilities.

The control structure developed in this paper has since been implemented in an individual with SCI [21]. That study was published as a companion article to the current study, and it focused on the practical implementation of these methods. The current study focuses on developing the methodologies, background, and development of the trajectory optimization and controllers. The current study also contributes to the generality of the findings in a way that is not possible with practical implementation. Implementation of this controller for use in daily life would require improvements in both modeling accuracy — possibly through the inclusion of more complicated muscle-force relationships including force-velocity relationships and activation dynamics — and optimization time — it is unfeasible to wait over a minute every time a person needs to reach. Additionally, some of the trajectories output by the optimization initially move away from the desired target (see Fig. 3) which may be undesirable for some tasks (consider eating). Further work with different optimization functions or weighting of the terms in (5) may be necessary for these tasks.

This simulation study has developed a data-driven trajectory planning method for successfully achieving FES-driven reaching motions. This novel method uses experimentally measured capabilities of an individual with SCI to find feasible reaching trajectories. With the right control structure — feedforward-feedback or MPC were found to be best in this study — this methodology should be practically implemented in individuals with SCI to improve FES-driven reaching.

## Supplementary Material

supp1-3272929

## References

[1] Ambrosini E , “The combined action of a passive exoskeleton and an EMG-controlled neuroprosthesis for upper limb stroke rehabilitation: First results of the RETRAINER project,” in Proc. Int. Conf. Rehabil. Robot. (ICORR), Jul. 2017, pp. 56–61. 10.1109/ICORR.2017.8009221 28813793

[2] Jagodnik KM, Thomas PS, Van Den Bogert AJ, Branicky MS, and Kirsch RF, “Training an actor-critic reinforcement learning controller for arm movement using human-generated rewards,” IEEE Trans. Neural Syst. Rehabil. Eng, vol. 25, no. 10, pp. 1892–1905, Oct. 2017. 28475063 10.1109/TNSRE.2017.2700395 PMC7523734

[3] Freeman CT, “Upper limb electrical stimulation using input-output linearization and iterative learning control,” IEEE Trans. Control Syst. Technol, vol. 23, no. 4, pp. 1546–1554, Jul. 2015.

[4] Sheffler LR and Chae J, “Neuromuscular electrical stimulation in neurorehabilitation,” Muscle Nerve, vol. 35, no. 5, pp. 562–590, 2007. 17299744 10.1002/mus.20758

[5] Downey RJ, Bellman MJ, Kawai H, Gregory CM, and Dixon WE, “Comparing the induced muscle fatigue between asynchronous and synchronous electrical stimulation in able-bodied and spinal cord injured populations,” IEEE Trans. Neural Syst. Rehabil. Eng, vol. 23, no. 6, pp. 964–972, Nov. 2015. 25350934 10.1109/TNSRE.2014.2364735

[6] Peckham PH and Knutson JS, “Functional electrical stimulation for neuromuscular applications,” Annu. Rev. Biomed. Eng, vol. 7, pp. 327–360, Aug. 2005. 16004574 10.1146/annurev.bioeng.6.040803.140103

[7] Jagodnik KM and van den Bogert AJ, “Optimization and evaluation of a proportional derivative controller for planar arm movement,” J. Biomech, vol. 43, no. 6, pp. 1086–1091, 2010. 20097345 10.1016/j.jbiomech.2009.12.017 PMC3853125

[8] Abreu J, Crowder DC, and Kirsch RF, “Deep reinforcement learning for control of time-varying musculoskeletal systems with high fatigability: A feasibility study,” IEEE Trans. Neural Syst. Rehabil. Eng, vol. 30, pp. 2613–2622, 2022. 36063517 10.1109/TNSRE.2022.3203970

[9] Razavian RS, Ghannadi B, Mehrabi N, Charlet M, and McPhee J, “Feedback control of functional electrical stimulation for 2-D arm reaching movements,” IEEE Trans. Neural Syst. Rehabil. Eng, vol. 26, no. 10, pp. 2033–2043, Oct. 2018. 29994402 10.1109/TNSRE.2018.2853573

[10] Klauer C , “Feedback control of arm movements using neuromuscular electrical stimulation (NMES) combined with a lockable, passive exoskeleton for gravity compensation,” Frontiers Neurosci, vol. 8, pp. 1–16, Sep. 2014. 10.3389/fnins.2014.00262 PMC4151235 25228853

[11] Pedrocchi A , “MUNDUS project: Multimodal neuroprosthesis for daily upper limb support,” J. Neuroeng. Rehabil, vol. 10, no. 1, p. 66, 2013. 23822118 10.1186/1743-0003-10-66 PMC3733825

[12] Ajiboye AB , “Restoration of reaching and grasping movements through brain-controlled muscle stimulation in a person with tetraplegia: A proof-of-concept demonstration,” Lancet, vol. 389, no. 10081, pp. 1821–1830, 2017. 28363483 10.1016/S0140-6736(17)30601-3 PMC5516547

[13] Wolf DN and Schearer EM, “Holding static arm configurations with functional electrical stimulation: A case study,” IEEE Trans. Neural Syst. Rehabil. Eng, vol. 26, no. 10, pp. 2044–2052, Oct. 2018. 30130233 10.1109/TNSRE.2018.2866226 PMC6284830

[14] Hasse BA, Sheets DEG, Holly NL, Gothard KM, and Fuglevand AJ, “Restoration of complex movement in the paralyzed upper limb,” J. Neural Eng, vol. 19, no. 4, Aug. 2022, Art. no. 046002. 10.1088/1741-2552/ac7ad7 PMC9284418 35728568

[15] Wolf DN, Hall ZA, and Schearer EM, “Model learning for control of a paralyzed human arm with functional electrical stimulation,” in Proc. IEEE Int. Conf. Robot. Autom. (ICRA), May 2020, pp. 10148–10154.

[16] Yamaguchi GT and Zajac FE, “Restoring unassisted natural gait to paraplegics via functional neuromuscular stimulation: A computer simulation study,” IEEE Trans. Biomed. Eng, vol. 37, no. 9, pp. 886–902, Sep. 1990. 2227975 10.1109/10.58599

[17] Popović D, Stein R, Oğuztöreli M, Lebiedowska M, and Jonić S, “Optimal control of walking with functional electrical stimulation: A computer simulation study,” IEEE Trans. Rehabil. Eng, vol. 7, no. 1, pp. 69–79, Mar. 1999. 10188609 10.1109/86.750554

[18] Sharma N, Mushahwar V, and Stein R, “Dynamic optimization of FES and orthosis-based walking using simple models,” IEEE Trans. Neural Syst. Rehabil. Eng, vol. 22, no. 1, pp. 114–126, Jan. 2014. 24122568 10.1109/TNSRE.2013.2280520

[19] Schearer EM and Wolf DN, “Predicting functional force production capabilities of upper extremity functional electrical stimulation neuroprostheses: A proof of concept study,” J. Neural Eng, vol. 17, no. 1, Feb. 2020, Art. no. 016051. 10.1088/1741-2552/ab68b3 31910397

[20] Blana D, Kirsch RF, and Chadwick EK, “Combined feedforward and feedback control of a redundant, nonlinear, dynamic musculoskeletal system,” Med. Biol. Eng. Comput, vol. 47, no. 5, pp. 533–542, May 2009. 19343388 10.1007/s11517-009-0479-3 PMC2971668

[21] Wolf DN and Schearer EM, “Trajectory optimization and model predictive control for functional electrical stimulation-controlled reaching,” IEEE Robot. Autom. Lett, vol. 7, no. 2, pp. 3093–3098, Apr. 2022.

[22] Schearer EM , “Semiparametric identification of human arm dynamics for flexible control of a functional electrical stimulation neuroprosthesis,” IEEE Trans. Neural Syst. Rehabil. Eng, vol. 24, no. 12, pp. 1405–1415, Dec. 2016. 26955041 10.1109/TNSRE.2016.2535348 PMC5205577

[23] Wolf DN and Schearer EM, “Evaluating an open-loop functional electrical stimulation controller for holding the shoulder and elbow configuration of a paralyzed arm,” in Proc. Int. Conf. Rehabil. Robot. (ICORR), Jul. 2017, pp. 789–794. 10.1109/ICORR.2017.8009344 28813916

[24] Polasek KH , “Stimulation stability and selectivity of chronically implanted multicontact nerve cuff electrodes in the human upper extremity,” IEEE Trans. Neural Syst. Rehabil. Eng, vol. 17, no. 5, pp. 428–437, Oct. 2009. 19775987 10.1109/TNSRE.2009.2032603 PMC2927980

[25] Hart RL, Bhadra N, Montague FW, Kilgore KL, and Peckham PH, “Design and testing of an advanced implantable neuroprosthesis with myoelectric control,” IEEE Trans. Neural Syst. Rehabil. Eng, vol. 19, no. 1, pp. 45–53, Feb. 2011. 20876029 10.1109/TNSRE.2010.2079952

[26] Wu G , “ISB recommendation on definitions of joint coordinate systems of various joints for the reporting of human joint motion—Part II: Shoulder, elbow, wrist and hand,” J. Biomech, vol. 38, no. 5, pp. 981–992, May 2005. 15844264 10.1016/j.jbiomech.2004.05.042

[27] Rasmussen CE and Williams CK, Gaussian Processes for Machine Learning, vol. 2, no. 3. Cambridge, MA, USA: MIT Press, 2006.

[28] Schearer EM , “Multi-muscle FES force control of the human arm for arbitrary goals,” IEEE Trans. Neural Syst. Rehabil. Eng, vol. 22, no. 3, pp. 654–663, May 2014. 24122573 10.1109/TNSRE.2013.2282903 PMC4034352

[29] Chadwick EK, Blana D, van den Bogert AJ, and Kirsch RF, “A real-time, 3-D musculoskeletal model for dynamic simulation of arm movements,” IEEE Trans. Biomed. Eng, vol. 56, no. 4, pp. 941–948, Apr. 2009. 19272926 10.1109/TBME.2008.2005946 PMC2971671

[30] Levinson DA and Kane TR, AUTOLEV—A New Approach to Multibody Dynamics BT—Multibody Systems Handbook, Schiehlen W, Ed. Berlin, Germany: Springer, 1990, pp. 81–102.

[31] Chadwick EK , “Real-time simulation of three-dimensional shoulder girdle and arm dynamics,” IEEE Trans. Biomed. Eng, vol. 61, no. 7, pp. 1–10, Jul. 2014. 10.1109/TBME.2014.2309727 PMC4068297 24956613

[32] Flash T and Hogan N, “The coordination of arm movements: An experimentally confirmed mathematical model,” J. Neurosci, vol. 5, no. 7, pp. 1688–1703, Jan. 1985. 4020415 10.1523/JNEUROSCI.05-07-01688.1985 PMC6565116

[33] Van Den Bogert AJ, Blana D, and Heinrich D, “Implicit methods for efficient musculoskeletal simulation and optimal control,” Proc. IUTAM, vol. 2, pp. 297–316, Jan. 2011. 10.1016/j.piutam.2011.04.027 PMC3217276 22102983

[34] Wachter A, “An interior point algorithm for large-scale nonlinear optimization with applications in process engineering,” Ph.D. dissertation, Dept. Chem. Eng., Carnegie Mellon Univ., Pittsburgh, PA, USA, 2002.

[35] Richter H, Advanced Control of Turbofan Engines. Cham, Switzerland: Springer, 2011.

[36] Schearer EM , “Evaluation of a semi-parametric model for high-dimensional FES control,” in Proc. 7th Int. IEEE/EMBS Conf. Neural Eng. (NER), Apr. 2015, pp. 304–307.

[37] Kirsch N, Alibeji N, and Sharma N, “Nonlinear model predictive control of functional electrical stimulation,” Control Eng. Pract, vol. 58, pp. 319–331, Jan. 2017.

[38] Bao X, Kirsch N, and Sharma N, “Dynamic control allocation of a feedback linearized hybrid neuroprosthetic system,” in Proc. Amer. Control Conf. (ACC), Jul. 2016, pp. 3976–3981.

[39] Sun Z, Bao X, Zhang Q, Lambeth K, and Sharma N, “A tube-based model predictive control method for joint angle tracking with functional electrical stimulation and an electric motor assist,” in Proc. Amer. Control Conf. (ACC), May 2021, pp. 1390–1395.

[40] Kocijan J, Murray-Smith R, Rasmussen CE, and Girard A, “Gaussian process model based predictive control,” in Proc. Amer. Control Conf., vol. 3, Jun. 2004, pp. 2214–2219.

[41] Boedecker J, Springenberg JT, Wulfing J, and Riedmiller M, “Approximate real-time optimal control based on sparse Gaussian process models,” in Proc. IEEE Symp. Adapt. Dyn. Program. Reinforcement Learn. (ADPRL), Dec. 2014, pp. 1–8.

[42] Zucker M , “CHOMP: Covariant Hamiltonian optimization for motion planning,” Int. J. Robot. Res, vol. 32, nos. 9–10, pp. 1164–1193, 2013.

[43] Cooman P, “Nonlinear feedforward-feedback control of an uncertain, time-delayed musculoskeletal arm model for use in functional electrical stimulation,” Ph.D. dissertation, Dept. Biomed. Eng., Case Western Reserve Univ., Cleveland, OH, USA, 2014.

