# Supplementary material for: Data-Driven Dynamic Motion Planning for Practical FES-Controlled Reaching Motions in Spinal Cord Injury
Source: IEEE Trans Neural Syst Rehabil Eng. Author manuscript; Available in PMC 2023 Jun 8. (PMC10248726; doi:10.1109/TNSRE.2023.3272929)
Supplement: supp1-3272929 [file NIHMS1900146-supplement-supp1-3272929.pdf]

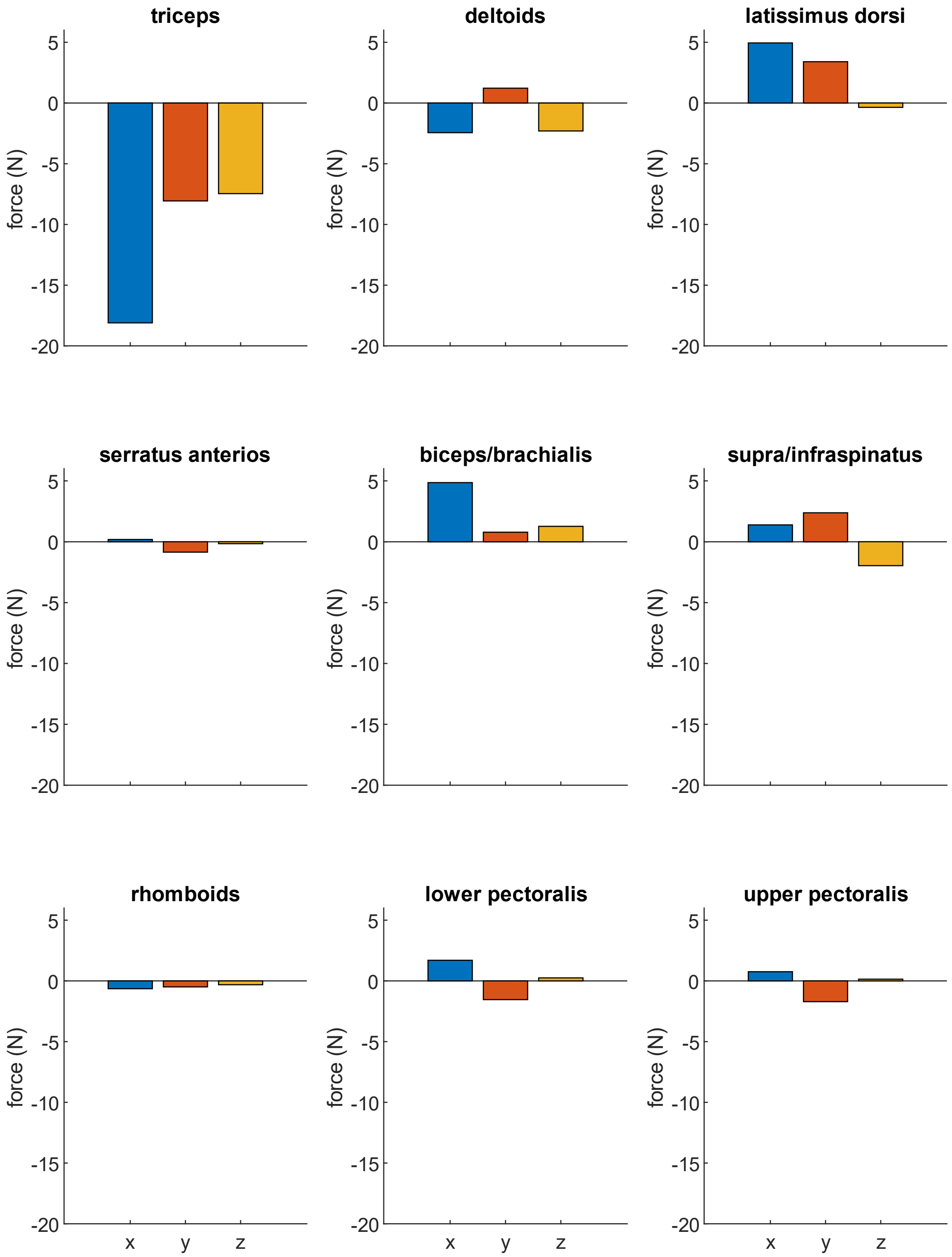

Fig. S1. Average forces recorded for each muscle group at maximum stimulation pulse-width during model identification at the starting point for each trajectory. The coordinate frame can be seen in Fig. 5(a).

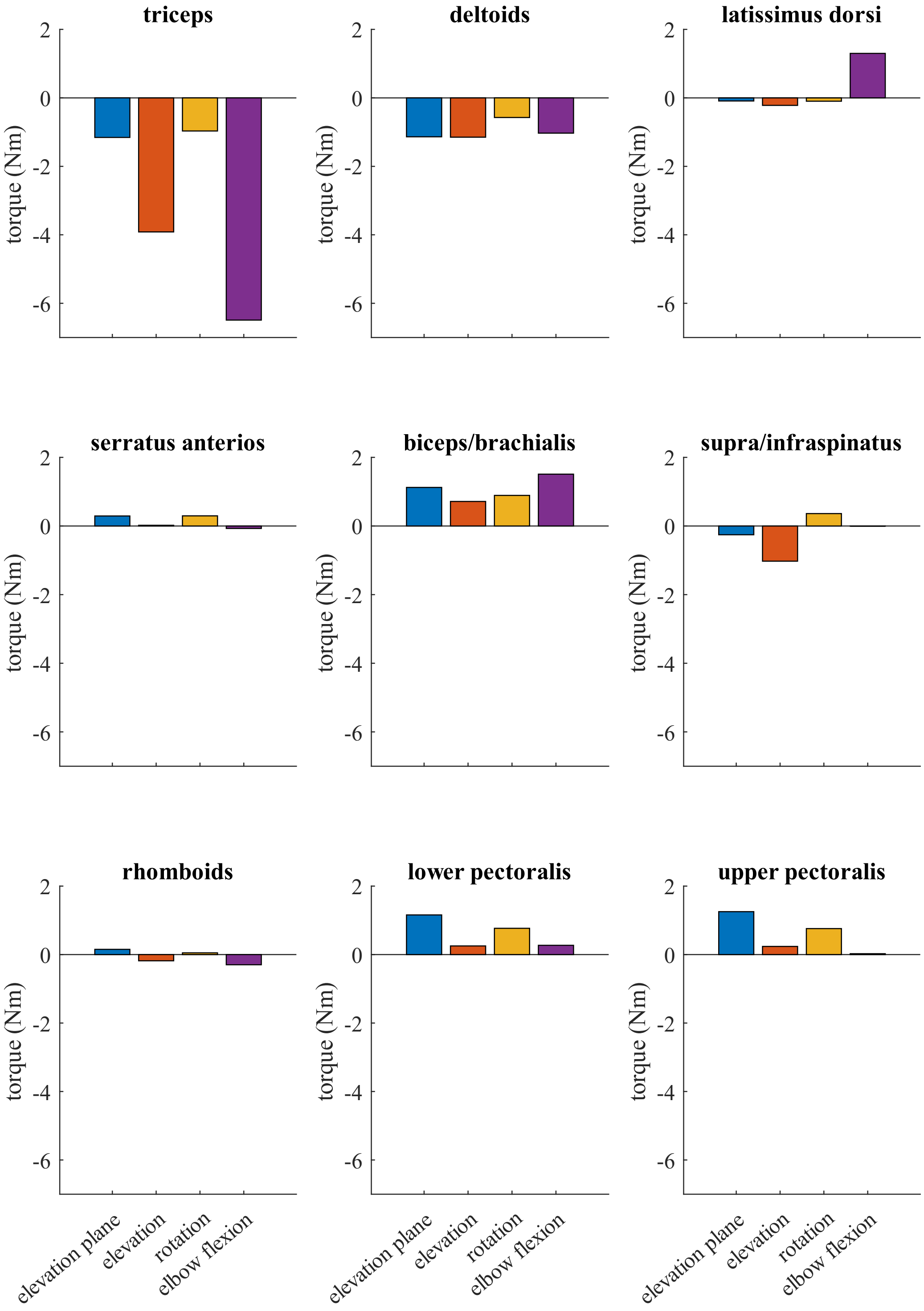

Fig. S2. Average joint torques for each muscle group at maximum stimulation pulse-width when calculated during model identification at the starting point for each trajectory.
